# Microfluidic-based fabrication, characterization and magnetic functionalization of microparticles with novel internal anisotropic structure

**DOI:** 10.1038/srep13060

**Published:** 2015-08-13

**Authors:** Yang Qiu, Fei Wang, Ying-Mei Liu, Wei Wang, Liang-Yin Chu, Hua-Lin Wang

**Affiliations:** 1 State Environmental Protection Key Laboratory of Environmental Risk Assessment and Control on Chemical Process, East China University of Science and Technology, Shanghai, 200237, P. R. China; 2 School of Chemical Engineering, State Key Laboratory of Polymer Materials Engineering, and Collaborative Innovation Center for Biomaterials Science and Technology, Sichuan University, Chengdu, Sichuan, 610065, P. R. China

## Abstract

Easy fabrication and independent control of the internal and external morphologies of core-shell microparticles still remain challenging. Core-shell microparticle comprised of a previously unknown internal anisotropic structure and a spherical shell was fabricated by microfluidic-based emulsificaiton and photopolymerization. The interfacial and spatial 3D morphology of the anisotropic structure were observed by SEM and micro-CT respectively. Meanwhile, a series of layer-by-layer scans of the anisotropic structure were obtained via the micro-CT, which enhanced the detail characterization and analysis of micro materials. The formation mechanism of the internal anisotropic structure may be attributed to solution-directed diffusion caused by the semipermeable membrane structure and chemical potential difference between inside and outside of the semipermeable membrane-like polymerized shell. The morphology evolution of the anisotropic structure was influenced and controlled by adjusting reaction parameters including polymerization degree, polymerization speed, and solute concentration difference. The potential applications of these microparticles in microrheological characterization and image enhancement were also proposed by embedding magnetic nanoparticles in the inner core.

Anisotropic microparticles have been receiving increased attention over the past years. The non-spherical shape and non-uniform chemical properties impart anisotropic microparticles the superiority to address inherent limitations of general isotropic microparticles^1^, which making them widely useful in diverse fields, such as biomedical engineering^2^,^3^, photonic crystals^4^,^5^, drug delivery^6^, emulsion stabilisation^7^, and micro mechanical elements^8^.

Basically, the most reported anisotropic microparticles are arising from their patchy surfaces or asymmetric shapes. For instance, since the first investigation reported in 1992^9^, studies about Janus microparticles^10^,^11^,^12^,^13^,^14^ have sprung up. With the distinct surface compositions building up the hemispheres, Janus microparticles bring us a mountain of possibilities in assembling complex material structure. Meanwhile, various microparticles having eye-dazzling non-spherical shapes also greatly enrich the arsenal of anisotropic materials. Particles with well-controlled shapes including rods, disks, ellipsoids, dumbbell-like, snowman-like, polygons^15^,^16^,^17^,^18^,^19^,^20^ were fabricated by miscellaneous synthetic methods. However, microparticles with internal anisotropic structure were rarely mentioned. Chen *et al.*^21^ fabricated magnetic hydrogel particles with uniform internal anisotropic features by flow focusing microfluidic method. Ge *et al.*^22^ reported a series of composite colloids embedding anisotropic structure through emulsion polymerization and phase separation. Besides, study about microparticles with internal anisotropic structure is nearly blank.

Conventionally, structure and morphology of microparticles are controlled by varying the compositions and properties of continuous or dispersed phases, the reaction temperature, time, or other triggering conditions^23^,^24^. Alternatively, geometric constraint, uniaxial stretching of initial template^25^,^26^, liquid phase deposition^27^,^28^, and microfabrication methods^29^,^30^,^31^ also have adjustment functions. Given the advantages including high continuity, reproducibility, scalability, and product controllability, microfluidic reactors were widely used to fabricate anisotropic microparticles with controlled sizes, shapes, and structures^32^,^33^,^34^. In microfluidic routes, the structure and shape of products could be tuned by physical confinement of microchannels^35^,^36^, solvent evaporation^37^, and adhesion energy variation^38^. Yet, the as-proposed methods would make the morphology and interface change of both internal and external part to be interactional, the independent formation and tuning of encapsulated anisotropic structure of microparticles still remain significantly challenging.

In this work, microparticles with novel internal anisotropic structure were developed for the first time. The microfluidic-based method to fabricate these microparticles was easy. Moreover, these novel microparticles have an internal anisotropic structure in a spherical overall shape. Water-in-oil-in-water (W/O/W) double emulsions from capillary microfluidics were used as the initial templates, and the solid shells of microparticles were obtained by UV photopolymerization. The formation mechanism was attributed to the osmotic pressure between the interior and exterior of the shell and the heterogeneous polymerization process of monomer. SEM and micro-CT were employed in the morphology characterization. A series of contrast experiments were carried out to verify the mechanism assumption and describe the controllability of the internal anisotropic structure. To investigate the potential application of these core-shell microparticles, superparamagnetic Fe_3_O_4_ nanoparticles were embedded into the cores. The magnetic responsiveness of the anisotropic structure enabled directivity for magnetic force lines, which would be further applied in micro-rheological study and magnetic line measurement.

## Results

### Fabrication process

**Figure 1:**
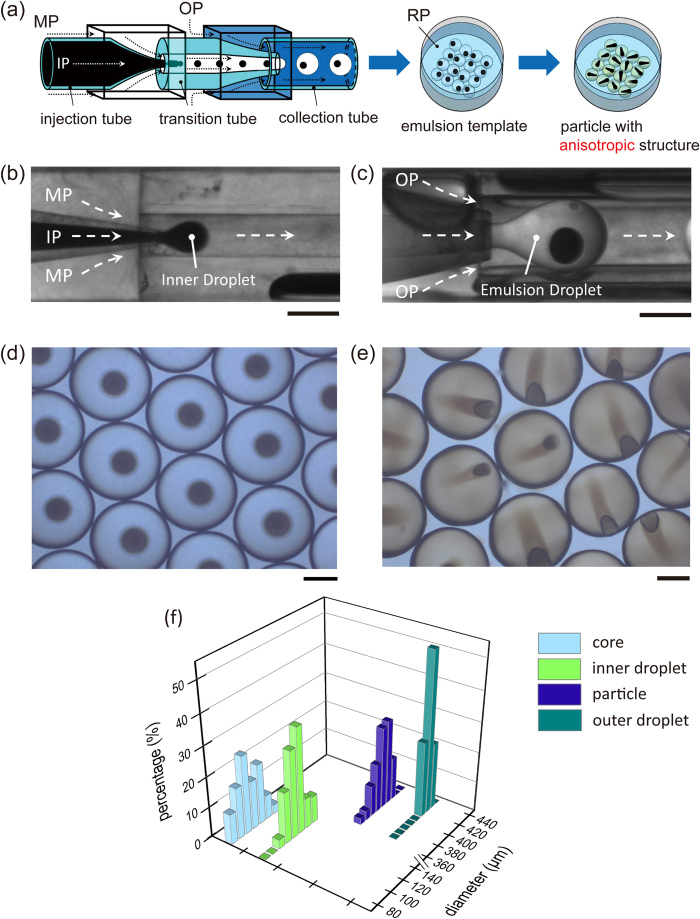
(**a**) Schematic of fabrication process of microparticles with internal anisotropic structures by microfluidic emulsification and off-chip UV photopolymerization. (**b**) Inner droplet fabrication in first tapered end. (**c**) Emulsion droplet fabrication in second tapered end. (**d**,**e**) Micrographs of template W/O/W emulsions (**d**) and microparticles with internal anisotropic structure (**e**). (**f**) Size distributions of emulsions and microparticles. Scale bars are 200 μm.

Figure 1a showed the schematic illustration of the fabrication process. For the template synthesis of our anisotropic microparticles, microfluidic device was used to prepare the monodisperse W/O/W multiple emulsions, each with a single inner droplet. Inner phase (noted as IP, water solution with surfactant and dye) was pumped into the injection tube and sheared into droplets in the first stage of the device by coaxial flow of middle phase (noted as MP, monomer comprising photo-initiator) as shown in Fig. 1b. The emulsions were subsequently emulsified in the second stage through coaxial flow of outer phase (noted as OP, water solution with surfactant) as shown in Fig. 1c. In both emulsification steps, the droplets immediately formed at the exit of the tapered end. This dripping mechanism ensured a highly monodisperse size distribution^39^. The precise control of flow rates (70~100 μL/h for IP, 1500~1800 μL/h for MP, 4000~5000 μL/h for OP) guaranteed all multiple emulsions embedding a single inner droplet. The prepared emulsions (Fig. 1d) were collected in a Petri-dish filled with receiving phase (noted as RP). After collecting enough emulsions, the Petri-dish was placed under a UV lamp to complete the off-chip photopolymerization process^40^ of the outer droplet. Generally, RP has a function in providing a liquid condition to ensure emulsions stable during the photopolymerization process and the compositions of RP should be totally the same as those of OP. But in our process, a certain amount of calcium chloride (1 mol/L) was dissolved in RP, which directly caused the formation of an unexpected internal anisotropic structure in the solidified microparticle (Fig. 1e). This unique anisotropic structure had an eccentric core, which could be attributed to the interfacial tension between the hydrophilic inner droplet and the hydrophobic monomer^41^. And more marvellously, a pointer-like anisotropic structure stretched from the core and inserted into the shell of miroparticles. Owe to the advantages of microfluidic routes, both the emulsions and microparticles had a very narrow size distribution, as shown in Fig. 1f. The coefficients of variance (defined as a standard deviation divided by the diameter of the droplets) were 1.38% and 3.86% for emulsions and particles respectively, which indicated the size distributions were highly monodisperse. The slight volume shrinkage of microparticles could be attributed to the density increase during the conversion from liquid to solid.

### Morphology observation

**Figure 2:**
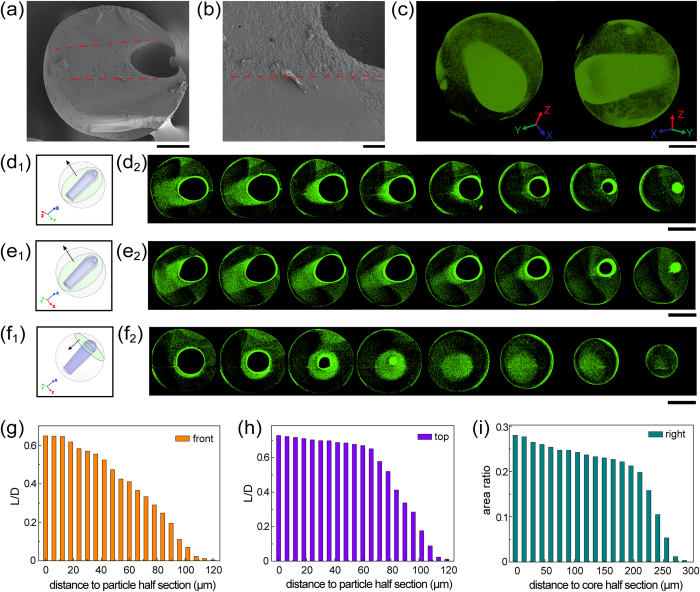
(**a**) Overall and (**b**) local amplification SEM image of microparticles with internal anisotropic structure. The red dash lines represent the boundary between the anisotropic structure and particle shell. (**c**) 3D model of single microparticle with internal anisotropic structure. (**d**_**1**_) ~ (**f**_**1**_) Schematic and (**d**_**2**_) ~ (**f**_**2**_) micro-CT image results of layer-by-layer scans for the “front”, “top” and “right” projective plane, respectively. (**g**) ~ (**h**) Variation in *L/D* as a function of distance to particle half section for the “front” and “top” projective plane. (**i**) Variation in area ratio as a function of distance to core half section for the “right” projective plane. Scale bars are 20 μm in (**b**), 100 μm in (**a**) and (**c**), 200 μm in (**d**_**2**_) ~ (**f**_**2**_).

The microscopic details of the internal anisotropic structure were primarily explored by SEM, as shown in Fig. 2a,b. From the SEM results, a sharp boundary between the anisotropic structure area and normal particle shell was found, as illustrated by the red dash line in the figures. Obviously, loose and porous structure filled the whole anisotropic zone, which was totally different from the surrounding smooth shell part. This morphology diversity indicated the anisotropic structure was not the shape change of core, but the result from the directed diffusion of the core encapsulating content.

In order to obtain more detailed information about the anisotropic structure, micro-CT technology was creatively introduced. The principle of this method is the detection of X-ray attenuation degree diversity when penetrating different materials or structures. Hence, by using this highly sensitive and precise characterization method, the 3D model and layer-by-layer scans of single particle could be obtained, as shown in Fig. 2c–f. The green colour displayed in the figures was added via the corresponding image process software, which would make different structures more obvious and discernible. In Fig. 2c, an all-around and nondestructive 3D model of single particle with anisotropic structure was presented. From the 3D perspective, the spatial region and volume of the anisotropic structure could be intuitively observed. Furthermore, for being more concrete and quantitative, series of layer-by-layer scans along the three axes were “cut”. To demonstrate the cut process, an x-y-z coordinate system and three projective planes were defined as followings: the “front” plane was parallel with the x-y plane. Similarly, the “top” plane was parallel with the x-z plane and “right” plane was parallel with the y-z plane. Then the virtual cutting was operated along the plane normal respectively. For the “front” and “top” projective plane, the scans were recorded from the particle half section to the margin of anisotropic structure, which were diagrammatized in Fig. 2d1,e1. Meanwhile, for the “right” projective plane, the scans were recorded from the core half section to the end of anisotropic structure, which were illustrated in Fig. 2f1. Commensurately, corresponding layer-by-layer scans were displayed in Fig. 2d2, e_2_ and f_2_. Based on these results, the relative length of anisotropic structure (defined as the ratio of the length of the structure to the maximal outer diameter of the microparticle, noted as *L/D*) and area ratio (defined as the ratio of the area of the structure to the maximal projected area of the microparticle) of every scanning section were counted and demonstrated in Fig. 2g–i. According to the statistic data, with the increasing distance from the core, *L/D* and cross-sectional area of the anisotropic structure tend to decrease, which means the anisotropic structure has a tapered pointer appearance. The space occupied by the anisotropic structure is about 240 × 240 × 300 μm and the volume of the anisotropic structure could be quantitatively calculated by calculus.

### Formation mechanism

**Figure 3:**
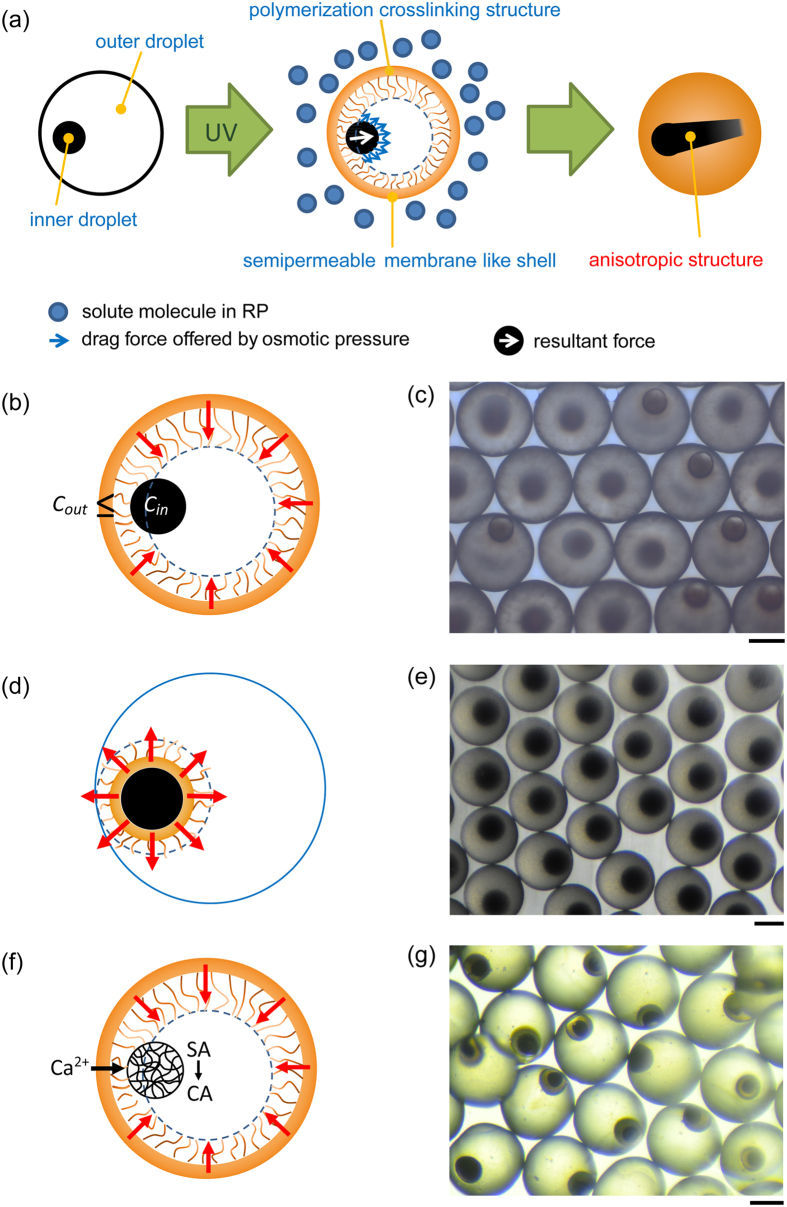
(**a**) Schematic of formation mechanism hypothesis. (**b**) Schematic and (**c**) fabricated microparticles of control experiments in condition of lower outside solution concentration. (**d**) Schematic and (**e**) fabricated microparticles of control experiments in condition of hydrophilic photoinitiator dissolved in IP. (**f**) Schematic and (**g**) fabricated microparticles of control experiments in condition of CA hydrogel constructed in core. Scale bars are 200 μm.

Based on the morphology observation, the formation mechanism of the internal anisotropic structure is hypothesized as follows and illustrated in Fig. 3a. The oil phase is converted into solid state from the outermost of the emulsion at the beginning of the photopolymerization, and then a densely reticular polymeric layer is formed and partly contacts the eccentric inner droplet. This cross-linked shell is similar with the structure of a semipermeable membrane and offers a passageway between core and outside solution. Meanwhile, CaCl_2_ solved in RP causes solution concentration difference between the interior of the core and the exterior of the semipermeable membrane-like shell. Under these conditions, the IP inside the core has a lower chemical potential than the RP outside of the shell, where osmotic pressure is formed^42^. Drag forces acting on the inner aqueous phase are offered by the osmotic pressure and oriented to outside of the shell. Given the eccentricity of the inner droplet, the cross-linked structure of the shell would partly encircle the inner droplet, restrain liquid diffusion in outward normal direction of the contacting camber surface, and favor inner liquid move in an opposite orientation. Integrated effects of the two key factors, the resultant force causes the morphology evolution of the internal phase and the internal anisotropic structure is obtained after the completion of the polymerization process.

In order to support the hypothesis, three control experiments were conducted. Schematics of these experiments are shown in Fig. 3b,d,f respectively. Firstly, emulsions were polymerized when IP solved equal or more amount of metal salt than RP (*C*_*in*_ ≥ *C*_*out*_), then common core-shell microparticles without internal anisotropic structure were synthesized (Fig. 3d). Compared with the initial formation process, higher chemical potential in the inner droplet was gained when the polymerization occurred in solution with lower concentration. No molecular exosmosis tendency of inner aqueous phase appeared. Subsequently, the internal structure would remain the same shape until the polymerization finished. This result indicates the importance of osmotic pressure in the inner structure evolution. Secondly, hydrophilic photoinitiator (V50 or Darocur 2959) dissolved in IP was used to replace hydrophobic photoinitiator previously used in MP. Similarly, no internal anisotropic structure was obtained (Fig. 3e). This experiment further provides powerful evidences for our hypothesis. Different photoinitiators cause the transfer of polymerization starting point. When hydrophilic photoinitiator was used, polymerization process began from the inner droplet and expanded from center to around. This “inside-out” polymerization would isolate the inner core from external solution throughout the whole process. The inside and outside solution exchange channel has no chance to be constructed, which hinders the formation of internal anisotropic structure. Thirdly, sodium alginate (SA) was added in IP. When the polymerization began, Ca^2+^ passed the shell and conducted cross-linking reaction with SA in the core. Then, calcium alginate (CA) hydrogel was constructed. The enclosed structure of hydrogel would eliminate the fluidity of IP, the liquid diffusion was blocked, and the internal anisotropic structure was disappeared. Combined with these three control experiments, the as-proposed mechanism hypothesis is fully proved and a clear explanation for the anisotropic structure formation is offered.

### Effects of solutes in RP

To quantitatively evaluate the optimal synthesis conditions and controllability of the anisotropic structure, two parameters, namely, yield of microparticles with internal anisotropic structure (defined as the ratio of the number of microparticles with internal anisotropic structure to the sum number of fabricated microparticles, noted as “*yield*” for short) and the above-mentioned *L/D*, were introduced. Since the additional CaCl_2_ dissolved in RP is the triggering condition for the anisotropic structure formation, the effect of solute in RP is primarily investigated. Various RP and IP solutions with different solute concentrations were prepared and coupled to obtain a series of concentration difference values (Δ*C* = *C*_*out*_ − *C*_*in*_) from 0.2 to 2 mol/L (detailed solution concentrations are listed in Table S1). Besides CaCl_2_, other metal salt solutes including CuCl_2_, NaI, NaCl, and Na_2_SO_4_ were selected to carry out contrast experiments.

**Figure 4:**
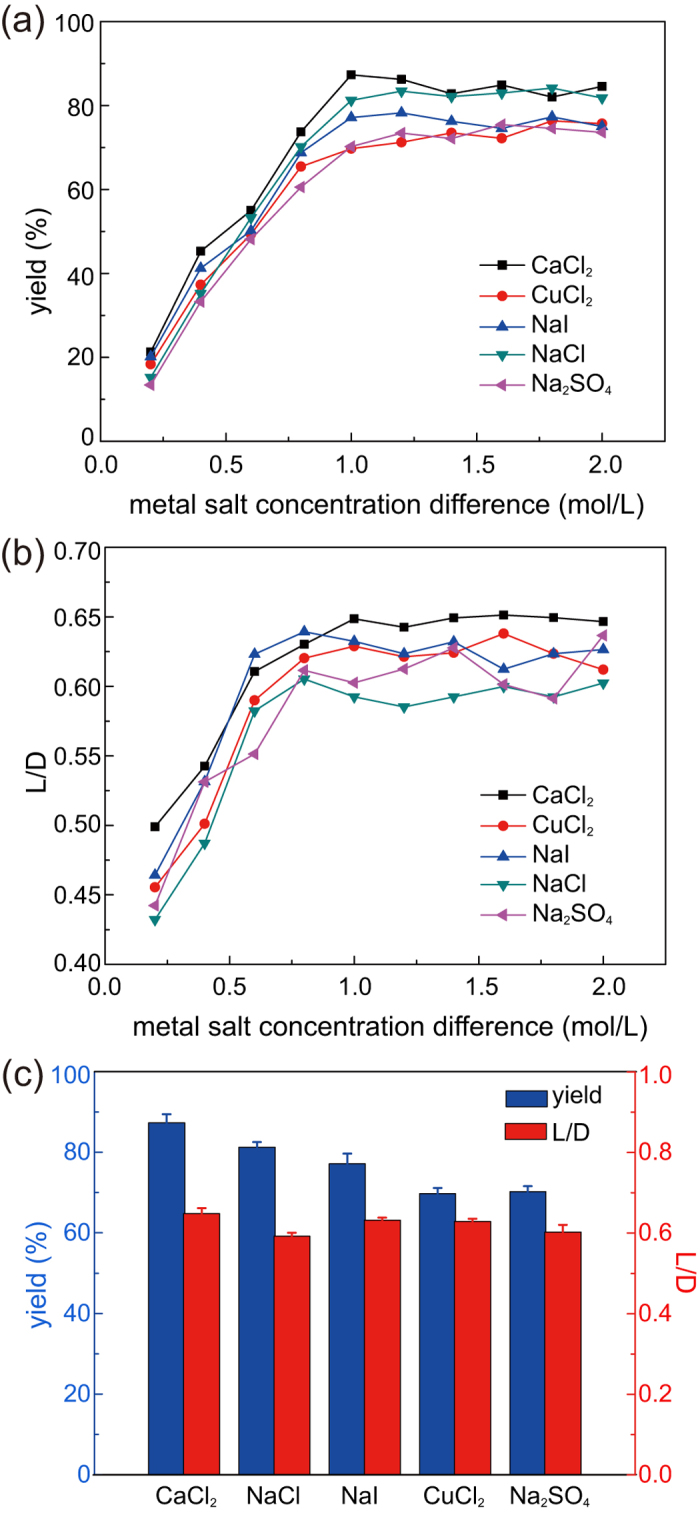
Variation in *yield* (**a**) and *L/D* (**b**) as a function of Δ*C* when solving 1 mol/L CaCl_2_ (black square), CuCl_2_ (red dot), NaI (blue triangle), NaCl (green triangle), and Na_2_SO_4_ (pink triangle) in RP solution. (**c**) Effect of different solutes on *yield* and *L/D* at Δ*C* = 1 mol/L.

With increasing Δ*C*, the inner core start to take morphology evolution. Figure 4a,b show in the range of 0 < Δ*C* < 1, both *yield* and *L/D* increase almost linearly with increasing Δ*C*, which is attributed to the osmotic pressure effect. Furthermore, both *yield* and *L/D* tend to be a fixed value when Δ*C* reaches over a threshold value approximately 1 mol/L. In addition, the types of solutes do not have a significant effect on either the *yield* or the *L/D* value through crosswise comparison between different solutes (Fig. 4c). Thus, RP-IP solute concentration difference is the major influence factor in the effect of RP solution on the internal anisotropic structure formation, whereas the solute type has a supporting function. Higher concentration difference brings stronger osmotic pressure, which eventually causes the morphology evolution.

### Effect of polymerization rate

Properties of RP are not the only factors affecting the anisotropic structure evolution. No anisotropic structure was found when the droplet in emulsion state (Fig. S1), which indicates the anisotropic structure formation is also associated with the photopolymerization reaction. To discuss the influence of photopolymerization process on the anisotropic structure, UV light intensity^43^ and photoinitiator concentration^44^ were selected to control the polymerization rate of monomer.

**Figure 5:**
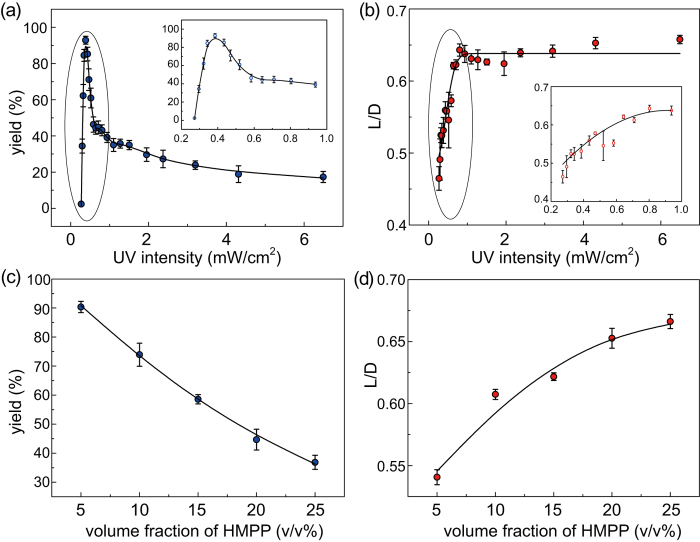
(**a**) Variation in *yield* as a function of *I.* (**b**) Variation in *L/D* as a function of *I*. Inserts in (**a**,**b**) show the plots within the range of 0.2 mW/cm^2^ <*I* <1 mW/cm^2^. (**c**) Variation in *yield* as a function of photoinitiator concentration. (**d**) Variation in *L/D* as a function of photoinitiator concentration.

UV light intensity (noted as *I*) was adjusted by changing the distance between UV lamp and Petri-dish (Fig. S2). Figure 5a shows that the *yield* increases with decreasing *I*, especially increases sharply after *I* < 1 mW/cm^2^ and reaches a maximum value (95.41%) at *I* = 0.4 mW/cm^2^. Then, a rapid decline appears in *yield* with further reducing of *I*, which is due to the large number of unpolymerized emulsions under the weak UV light intensity. Meanwhile, *L/D* does not significantly decrease until *I* reduces under 1 mW/cm^2^. After *I* < 1 mW/cm^2^, *L/D* shows a contrary tendency to the *yield* law, which decreases significantly as function of reducing value of *I* (Fig. 5b). In particular, *I* = 1 mW/cm^2^ is a turning value point for obvious relationship of either *yield* or *L/D* with *I*.

Monomers comprising 5%, 10%, 15%, 20%, and 25% (v/v) HMPP were individually prepared to control the polymerization rate in an alternative approach. Figure 5c indicates that *yield* is reversely correlated with HMPP concentration. By contrast, *L/D* has a positive correlation with HMPP concentration, as shown in Fig. 5d. Briefly, UV light intensity (especially in the range of 0.4 mW/cm^2^ <*I* <1 mW/cm^2^) and photoinitiator concentration has similar effects on *yield* and *L/D* of the anisotropic structure. The same influence rule of these two factors proves that the polymerization rate of monomer has inhibiting effect on *yield*, but facilitating effect on relative length of the anisotropic structure. One of the optimal fabrication conditions of micropartilces with anisotropic structure is *I* = 0.4 mW/cm^2^.

### Effect of monomer conversion rate

**Figure 6:**
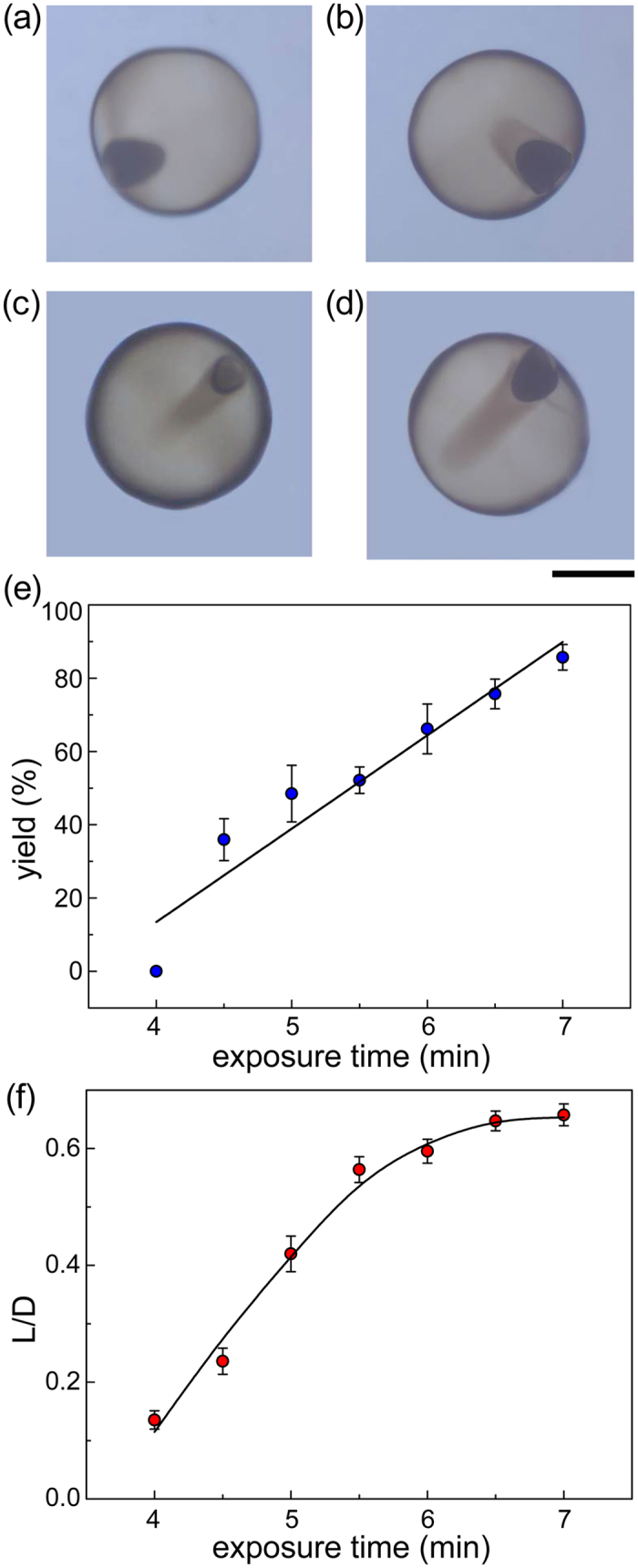
(**a**–**d**) Morphology evolution of the internal anisotropic structure at exposure time of 4 (**a**), 5 (**b**), 6 (**c**) and 7 (**d**) min, respectively. (**e**) Variation in *yield* as a function of exposure time. (**f**) Variation in *L/D* as a function of exposure time. Scale bar is 200 μm.

For polymerization process, monomer conversion rate is another important factor, which could be controlled by the exposure time of emulsions under UV light^45^. In this work, it was observed that the polymerization process was not carried out via emulsions until exposed under UV light for 4 min at *I* = 0.4 mW/cm^2^, and the polymerization was completed after exposing emulsions under UV for 7 min. During this reaction time period, the anisotropic structure has a visually synchronous change with polymerization degree (Fig. 6a–d), and both *yield* and *L/D* have a positive correlation with exposure time (Fig. 6e,f). This finding further explains that the anisotropic structure formation is affected by monomer conversion rate. In particular, the change of *L/D* is the most significant in all of the four experiments (0.1–0.7 for this experiment, 0.4–0.7 for the other three). This result indicates that the morphology of the anisotropic structure is mainly controlled by monomer conversion rate. The as-proposed results also indicate that the polymerized shell is a pacing factor for anisotropic structure formation, which proves our mechanism hypothesis on the semipermeable membrane-like shell concept.

### Magnetic functionalization

**Figure 7:**
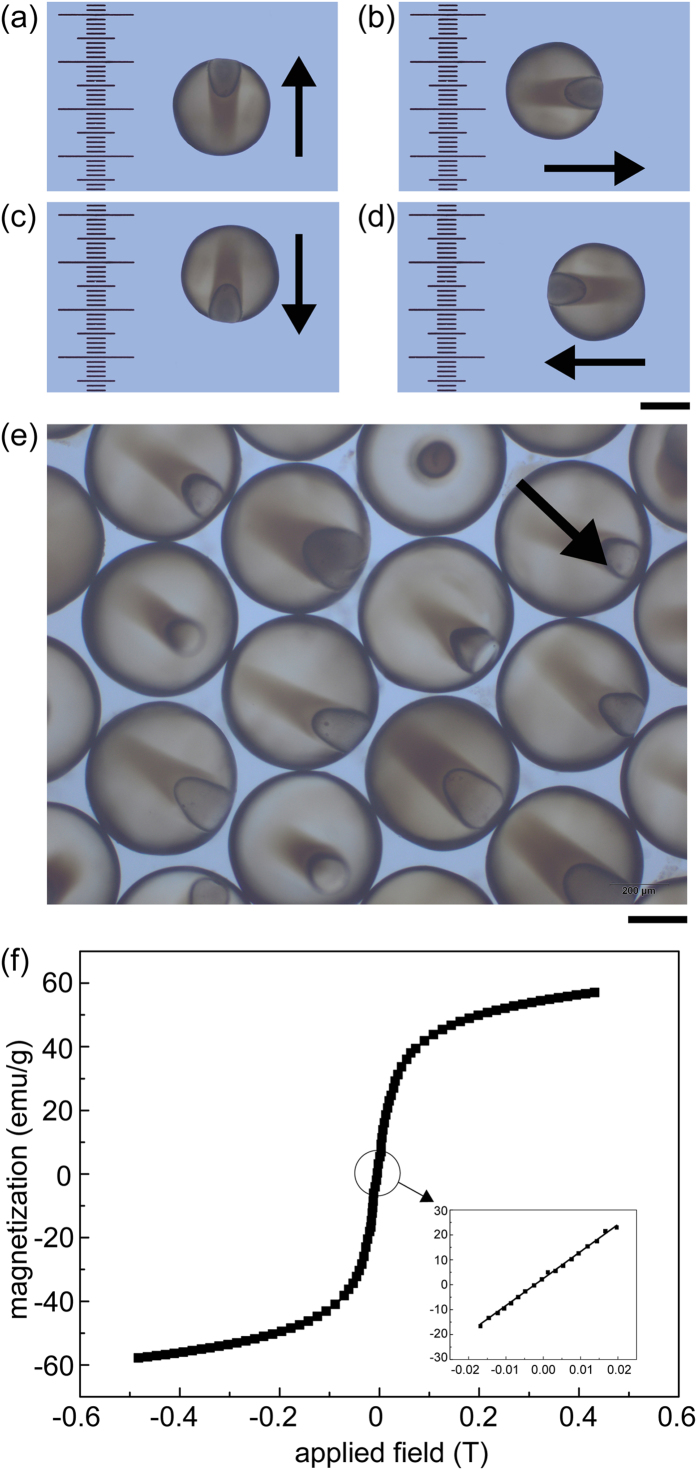
(**a**–**d**) Response of single microparticle with internal anisotropic structure to rotated magnetic field. (**e**) Response of batch of microparticles to an external magnetic field. Arrows indicate field directions. Scale bars are 200 μm. (**f**) Field-dependent magnetization curve of microparticles at 298 K. Insert illustrates the expanded magnetization curves in a lower applied field display without hysteresis.

Magnetization is introduced to explore morphological advantages of the anisotropic structure and exploit its potential applications. The prepared Fe_3_O_4_ magnetic nanoparticles were uniformly dispersed in IP and embedded in the cores after polymerization, giving the microparticles superparamagnetic behavior. To obtain better magnetic response, larger cores were fabricated by adjusting the flow rates in microfluidics. By placing single microparticle in a magnetic field and then rotating the field, the particle acquired a dipole moment and rotates freely, such that its anisotropic structure aligned with the direction of the field without physical translation along the field plane (Fig. 7a–d). Given the ability to be manipulated remotely and sensitive response to the external field, a batch of microparticles exhibited a collectively directional preference to an external magnetic field arising from the shape asymmetry of the anisotropic structure (Fig. 7e). With the precise response, directivity and tracing ability, these microparticles could serve as probes for microrheological characterization of complex fluids and biomaterials^21^, and as contrast enhancers in magnetic imaging^46^, Fig. 7f illustrates that the microparticles have a superparamagnetic behavior. No hysteresis is found at low field strength, which is another important feature for potential applications where no memory of magnetization after removing external magnetic fields is highly desirable.

## Discussion

In summary, microparticles with internal anisotropic structure have been introduced. An approach based on co-flow capillary microfluidic emusificaiton and off-chip UV photopolymerization was developed to fabricate these novel microparticles in monodisperse dimensions. The interfacial morphology of the anisotropic structure was observed by SEM. Creatively, nondestructive 3D model and layer-by-layer scans obtained via micro-CT technology quantitatively described the spatial morphology of the internal anisotropic structure and deepened the characterization methods for micro material. Chemical potential difference offered by semipermeable membrane-like polymerized shell and solute concentration difference between the inner core and the outer shell favor the anisotropic structure formation, which was verified by three control experiments about concentration difference and polymerization starting point. The effects of various influence factors on yield and morphology of the anisotropic structures were systematically studied. Through a group of contrasting experiments, the concentration and composition of RP solution, the polymerization speed and conversion rate of monomer were found to have influences on the anisotropic structure parameters. The morphology of the anisotropic structure can be widely controlled. Besides, the optimal reaction conditions including the most appropriate RP solute (CaCl_2_), concentration difference (Δ*C* = 1 mol/L), UV intensity (*I* = 0.4 mW/cm^2^), and photoinitiator dosage (5%) have been confirmed. Moreover, these microparticles were functionalized by uniformly embedding magnetic nanoparticles in cores. The magnetized microparticles showed excellent remote and precise controllability by an external magnetic field. A batch of microparticles could constitute a display panel for magnetic field line direction, which has promising applications in microrheological study and magnetic image enhancement.

## Methods

### Materials

Ethyleneglycol dimethacrylate (EGDMA) (Sigma-Aldrich) was used as the base monomer solvent for oil phase and 2-Hydroxy-2-methylpropiophenone (HMPP) (Sigma-Aldrich) were used as the base monomer solvent and photoinitiator for oil phase, respectively. Pluronic F-127 (Sigma-Aldrich) and polyglycerol polyricinoleate (PGPR 90) (Danisco, Denmark) were used as the surfactants for aqueous phase and oil phase, respectively. All other reagents were of analytical grade and used as received. Aqueous solution (5 mL) with Pluronic F-127 [(0.05 g, 1% (w/v)], carbon black [(0.05 g, 1% (w/v)], and glycerol [(0.25 g, 5% (w/v)] were used as IP. Monomer EGDMA (5 mL) comprising PGPR 90 [(0.25 g, 5% (w/v)] and HMPP (0.05 mL, 1% (v/v)) was used as MP. The components of OP (20 mL) were the same as those of IP but without the dye. Aqueous solution containing 1 mol/L calcium chloride (CaCl_2_), 5% (w/v) glycerol, and 1% (w/v) Pluronic F-127 was used as RP.

### Microfluidic device

The capillary microfluidic device comprised two main stages^47^. The first stage includes an injection tube with a tapered end, which was inserted into the transition tube. The other end of the transition tube was also tapered and inserted into a continuous collection tube to constitute the second stage. Both cylindrical capillary tubes were centered within a larger square capillary. Alignment was ensured by matching the outer diameters of the cylindrical tubes to the inner side lengths of the square capillary. All the cylindrical capillary tubes were tapered by a micropuller (Narishige) and then adjusted by a microforge (Narishige). The square capillary was used as received (Vitrocom). Figure S3 and Table S2 show the detailed structure and size data.

### Preparation of magnetic nanoparticles

Magnetic nanoparticles were prepared by the co-precipitation method reported by Massart^48^. Briefly, hydrochloric acid (4 mL) and FeCl_2_·4H_2_O (7.2 g) were dissolved into 20 mL of deionized water. The resultant solution was mixed with 29 mL of 27 wt% FeCl_3_·6H_2_O aqueous solution. Then, 47 mL of deionized water was added to obtain up to 100 mL of solution. The addition of 40 mL of ammonia immediately turned the solution to black, which indicates that Fe_3_O_4_ nanoparticles were synthesized. The obtained nanoparticles were separated from the solution by a magnet and then redispersed in 90 mL of tetramethylammonium hydroxide (TMA) (1 mol/L) after removing the excess supernatant liquor. The TMA solution comprising magnetic nanoparticles was agitated at 300 rpm overnight to obtain a uniform ferrofluid. Finally, 1% (w/v) ammonium persulfate (0.9 g) was dissolved in the resultant solution to enhance the emulsibility.

### Micro-CT observation

Single particle was fixed on the top of a glass rod by double-sided foam tape. Then the glass rod was clamped on the holder of micro-CT device (phoenix nanotom m X-ray CT, GE). The scanning process was operated with the voltage of 80 kV, current of 100 μA, detection time of 100 min. The voxel size of results (3D model and layer-by-layer scans) was 1.25 μm. The modeling, processing and measurement of the images were achieved by corresponding image software (Volume Graphics, GE).

### SEM observation

SEM images of EGDMA microparicles were obtained by field-emission scanning electron microscopy (NOVA Nano SEM450, FEI). Microparticles were in dry-state for the SEM characterization. The preparation process of samples included three steps. First, microparticles were dried by vacuum-heating method. Second, microparticles were cut in half by blade to obtain the cross-section. In the cutting process, microparticles were fixed by glass slide and cover glass, which could ensure the cutting precision. After cutting, microparticles with lossless facet were selected by optical microscope to conduct the following step. Third, Pt surface spraying was done to improve the electrical conductivity of the microparticles.

### Other characterizations

Multiple emulsion formation was observed under an inverted optical microscope (Ti-S, Nikon) and recorded by a high-speed digital camera (N4-S3, IDT). Optical microscopic images of the emulsions and microparicles were obtained by using an inverted optical microscope with a CCD camera (Go-5, Qimaging). The size distribution, yield and relative length of the internal anisotropic structures were determined based on their optical micrographs using automatic analytic software (Image Pro Plus, Media Cybernetics). Magnetic response of single microparticle was observed on a glass side with micrometers (div = 2 μm) as a reference frame. A cuboid-shaped magnet (size: 500 mm × 200 mm × 200 mm) with a maximum magnetic intensity of 1.5 T was used to create a rotated magnetic field. The magnetization curve of the microparticles was measured by a vibrating sample magnetometer (Lakeshore 7407).

## Additional Information

**How to cite this article**: Qiu, Y. *et al.* Microfluidic-based fabrication, characterization and magnetic functionalization of microparticles with novel internal anisotropic structure. *Sci. Rep.* **5**, 13060; doi: 10.1038/srep13060 (2015).

## Supplementary Material

Supplementary Information
