# Supplementary material for: Microfluidic-based fabrication, characterization and magnetic functionalization of microparticles with novel internal anisotropic structure
Source: Sci Rep. 2015 Aug 13;5:13060. doi: 10.1038/srep13060 (PMC4535034; doi:10.1038/srep13060)
Supplement: Supplementary Information [file srep13060-s1.doc]

Supplementary Information

**Microfluidic-based fabrication, characterization and magnetic functionalization of microparticles with novel internal anisotropic structure**

Yang Qiu^1^, Fei Wang^1^, Ying-Mei Liu^2^, Wei Wang^2^, Liang-Yin Chu^2^ & Hua-Lin Wang^1*^

^1^State Environmental Protection Key Laboratory of Environmental Risk Assessment and Control on Chemical Process, East China University of Science and Technology, Shanghai, 200237, P. R. China

^2^State Key Laboratory of Polymer Materials Engineering, and Collaborative Innovation Center for Biomaterials Science and Technology, Sichuan University, Chengdu, Sichuan, 610065, P. R. China

* Corresponding authors

H.L.W.: E-mail address: wanghl@ecust.edu.cn, Tel: +86-21-6425 2748, Fax: +86-21-6425 1894

***Supplementary Table S1.* *Metal salt concentration of IP and RP and the various concentration differences***

| Group 1 | |  | Group 2 | | Δ*C* (mol/L) |
| --- | --- | --- | --- | --- | --- |
| *C_in_* (mol/L) | *C_out_* (mol/L) |  | *C_in_* (mol/L) | *C_out_* (mol/L) |
| 0 | 2 |  | 1 | 3 | 2 |
| 0 | 1.8 |  | 1 | 2.8 | 1.8 |
| 0 | 1.6 |  | 1 | 2.6 | 1.6 |
| 0 | 1.4 |  | 1 | 2.4 | 1.4 |
| 0 | 1.2 |  | 1 | 2.2 | 1.2 |
| 0 | 1 |  | 1 | 2 | 1 |
| 0 | 0.8 |  | 1 | 1.8 | 0.8 |
| 0 | 0.6 |  | 1 | 1.6 | 0.6 |
| 0 | 0.4 |  | 1 | 1.4 | 0.4 |
| 0 | 0.2 |  | 1 | 1.2 | 0.2 |


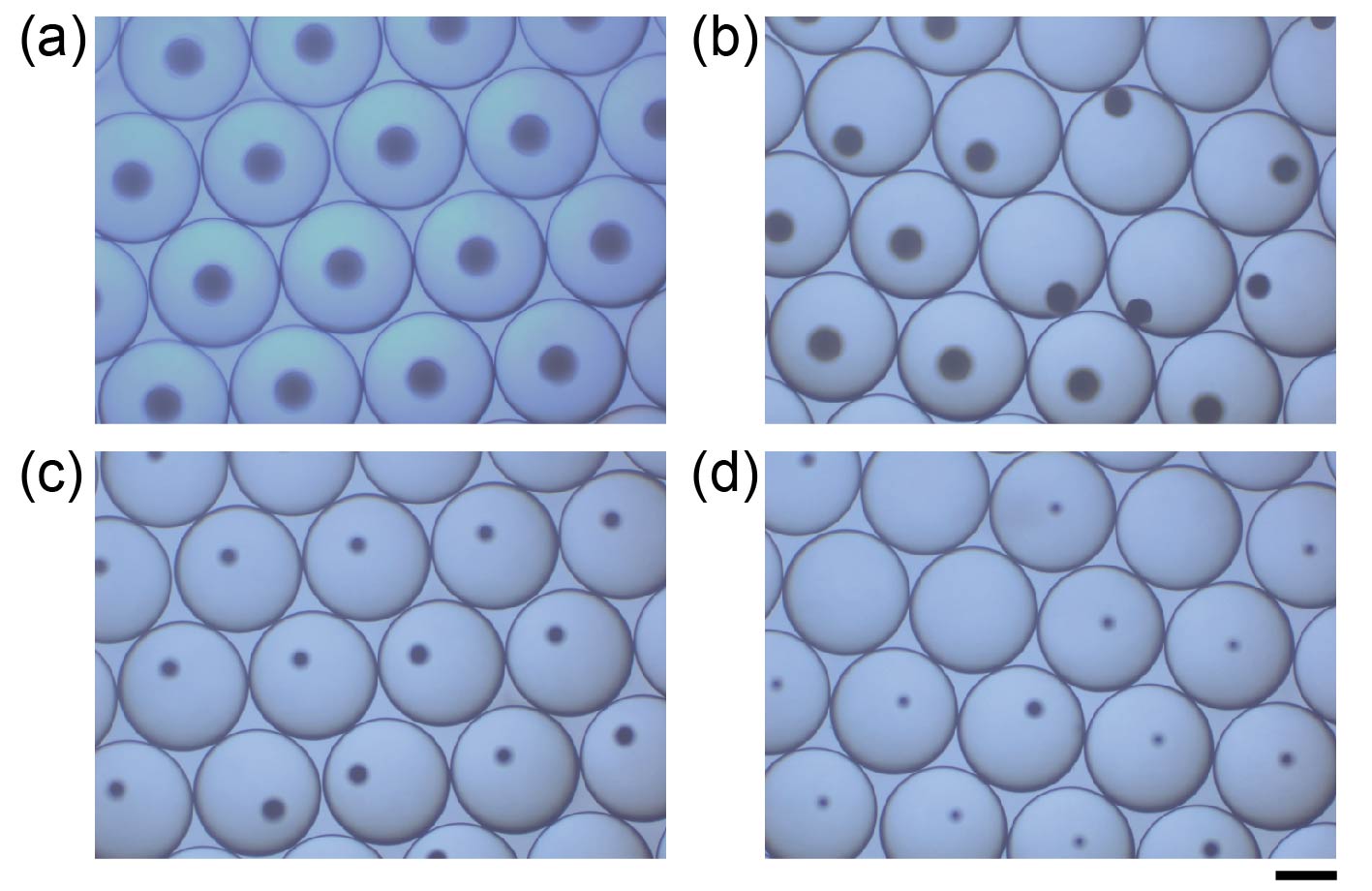


***Supplementary Figure S1.*** *Emulsion droplets saved in 1mol/L CaCl_2_ RP solution for (a) 0min, (b) 10min, (c) 30min, and (d) 60min. No pointer-like structure was found, the only change was the inner droplets were shrinking over time due to the instability caused by the density difference, lack of surfactant or some other reasons. Scale bar is 200μm.*

**
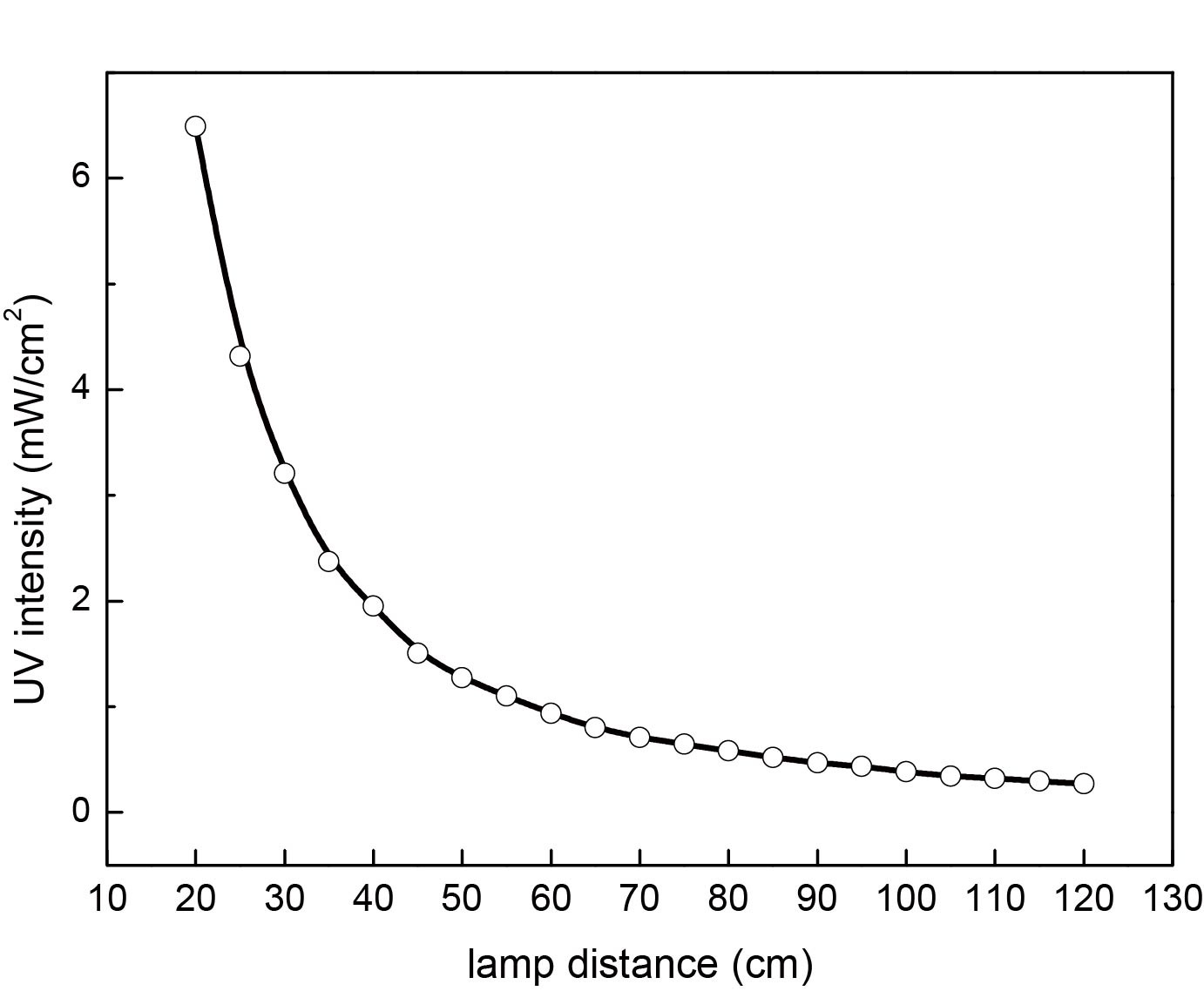
**

***Supplementary Figure S2.*** *Variation in UV light intensity (I) as a function of distances between lamp and petri dish, measured by UV radiation meter.*

**
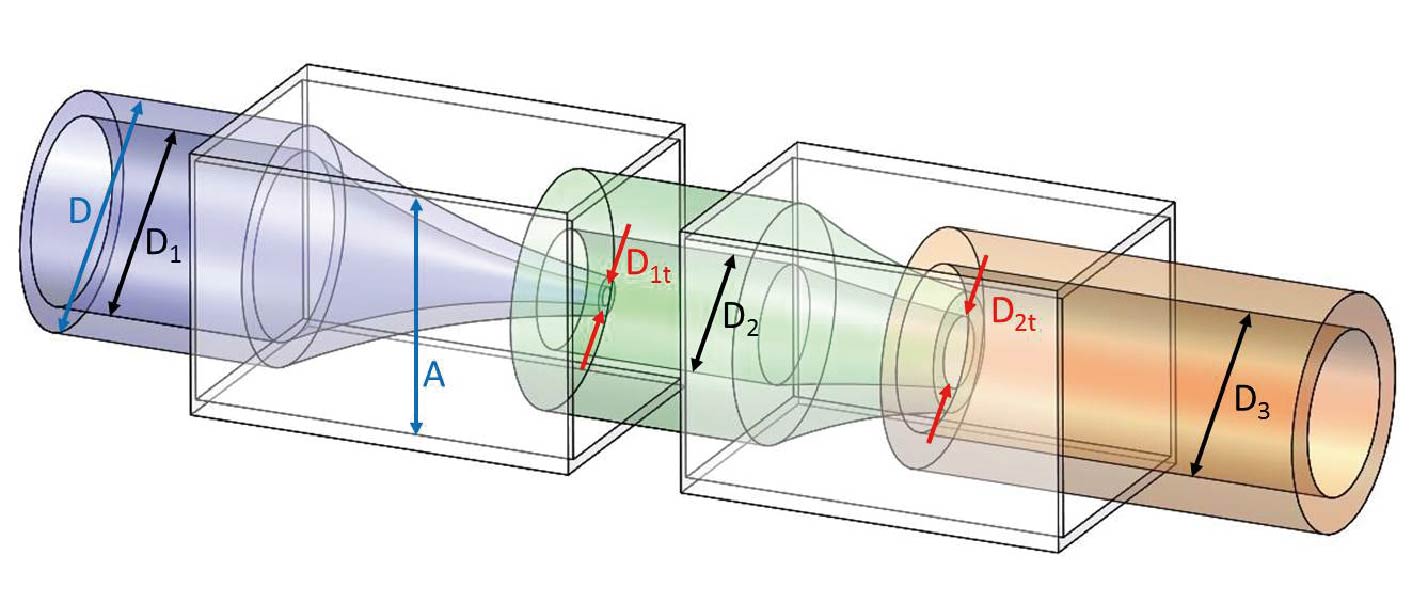
**

***Supplementary Figure S3.*** *Structure diagram of microfluidic device. D_1_, D_2_, D_3_ is the inner diameters of injection tube, transition tube and collection tube respectively. D_1t_, D_2t_ is the inner diameters of tapered ends of injection tube and transition tube respectively. D is the outer diameter of cylinder tube. A is the inner side length of square tube.*

***Supplementary Table S2.* *Detailed size data of microfluidic device in Figure S1***

| Dimension | Size (μm) |
| --- | --- |
| D_1_ | 580 |
| D_1t_ | 40 |
| D_2_ | 150 |
| D_2t_ | 100 |
| D_3_ | 500 |
| D | 990 |
| A | 1000 |
